# Clinical Characteristics of Idiopathic Granulomatous Mastitis in a Hispanic Border Population: A Case Series and Literature Review

**DOI:** 10.7759/cureus.56453

**Published:** 2024-03-19

**Authors:** Percy M Thomas, Laura R Uribe, Aminadab G Flores, Emilia Dulgheru

**Affiliations:** 1 Internal Medicine, Doctors Hospital at Renaissance, University of Texas Rio Grande Valley, Edinburg, USA; 2 Internal Medicine, Doctors Hospital at Renaissance, Edinburg, USA; 3 Rheumatology, Doctors Hospital at Renaissance, Edinburg, USA

**Keywords:** case report series, mastitis, idiopathic mastitis in hispanic female, autoimmune condition of breast, idiopathic granulomatous mastitis (igm)

## Abstract

Idiopathic granulomatous mastitis (IGM) is an autoimmune condition of the breast that is commonly encountered in women of non-white ethnicity such as Southeast Asians, Middle Easterners, and Hispanics. This condition often presents as a painful breast mass, and many patients undergo invasive diagnostic procedures or surgical excision, which can lead to disfiguring scars. Early recognition and prompt treatment with immunosuppressive medications can prevent invasive workups and management. Although previously thought to require an exclusively surgical approach, it now prompts interdisciplinary management. In this context, we present a case series of patients with IGM in a Hispanic population of South Texas.

## Introduction

Idiopathic granulomatous mastitis (IGM) is an inflammatory condition of the breast that has historically posed diagnostic challenges and is associated with a lack of consensus regarding management strategies. While various autoimmune diseases can occasionally target the breast, IGM is an autoimmune condition specific to the breast [1]. There is 12 times more predominance in women of Hispanic, Middle-Eastern, and Southeast Asian origin [2].

Multiple risk factors have been associated with IGM, including but not limited to, elevated prolactin level [3], foreign body reaction [3], breast trauma, contraceptive use, lactation, and breastfeeding weaning habits [4]. Additionally, autoimmune conditions, such as Wegener's granulomatosis, giant cell arteritis, and α1-antitrypsin deficiency [5], can increase the risk of developing autoimmune mastitis. It is essential to differentiate IGM from mimickers such as periductal mastitis [6], cystic neutrophilic mastitis [7], Corynebacterium kroppenstedtii [8], and tuberculous mastitis [9].

The clinical presentation of IGM varies among patients and can range from a mass that grows over time to unilateral painful mass, skin induration, skin ulceration, and areolar retraction to fistula formation [10]. Imaging tests, such as ultrasonography, MRI, or mammography, may reveal non-specific features in most cases, leading to biopsy. Histopathology may reveal non-necrotizing granulomas and organized microabscesses with multi-nucleated giant cells, lymphocytes, plasma cells, and epitheloid histiocytes [11].

The current knowledge of IGM as being an inflammatory, auto-immune disease has diverted the patient's care from surgical to medical treatment, specifically seeking rheumatological care. With this case series, we hope to provide insight into the clinical presentations of IGM and propose non-invasive management of IGM through a multispecialty approach.

## Case presentation

Method

We conducted a retrospective case series at an outpatient Rheumatology department between May 2022 and May 2023. The study identified five patients who were diagnosed with granulomatous mastitis. We analyzed their clinical presentation, demographic data, pathology findings, and management modalities upon chart review. All patients were referred to Rheumatology by a breast surgeon after undergoing a biopsy for breast mass evaluation. Patients were separated based on the treatment modality used and clinical outcome.

The clinical characteristics of the IGM/breast mass determined treatment modalities. These included antibiotic + COX-2 inhibitor, methotrexate (MTX) alone, MTX plus steroid therapy, or high-dose steroid only. Patients were followed for an average of six months. Complete remission was considered with treatment success, partial remission was considered when complete symptom resolution was not achieved.

**Figure 1:**
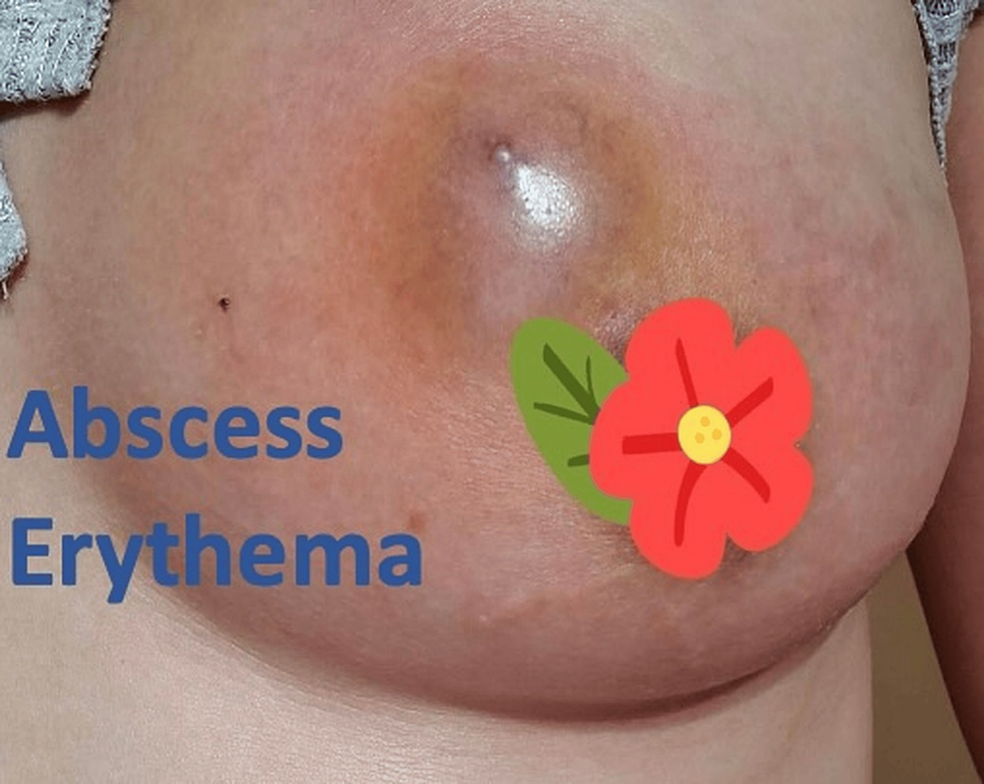
Case 5 breast examination showing an abscess with erythema

**Figure 2:**
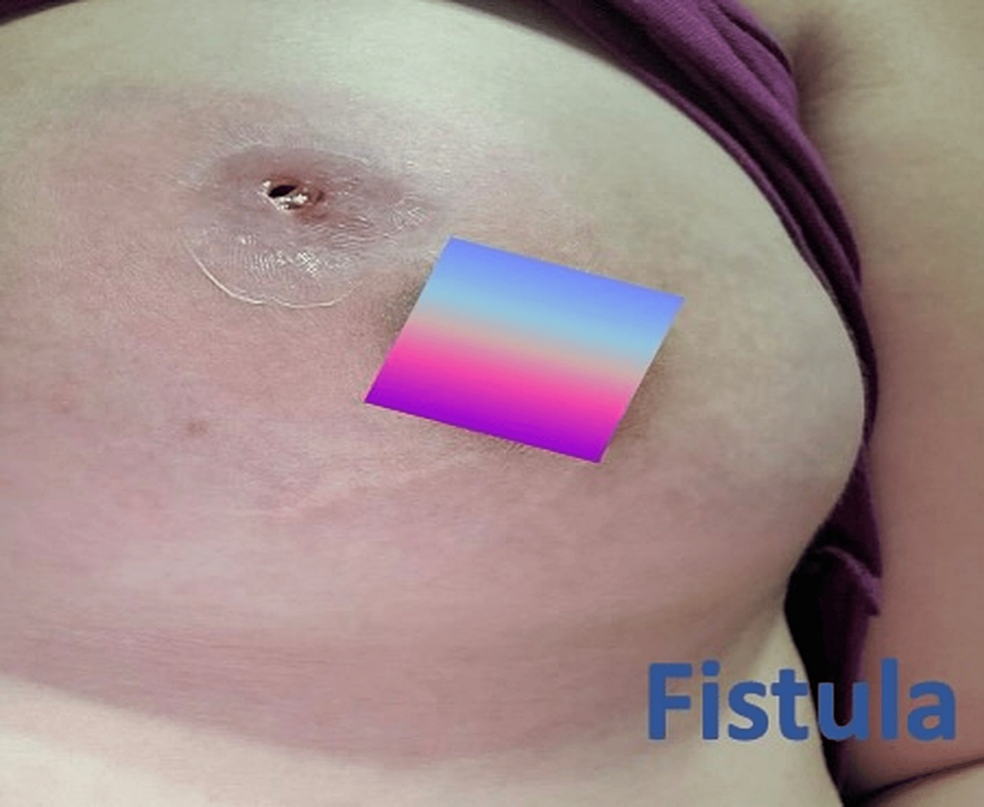
Case 5 breast examination showing fistula formation

**Figure 3:**
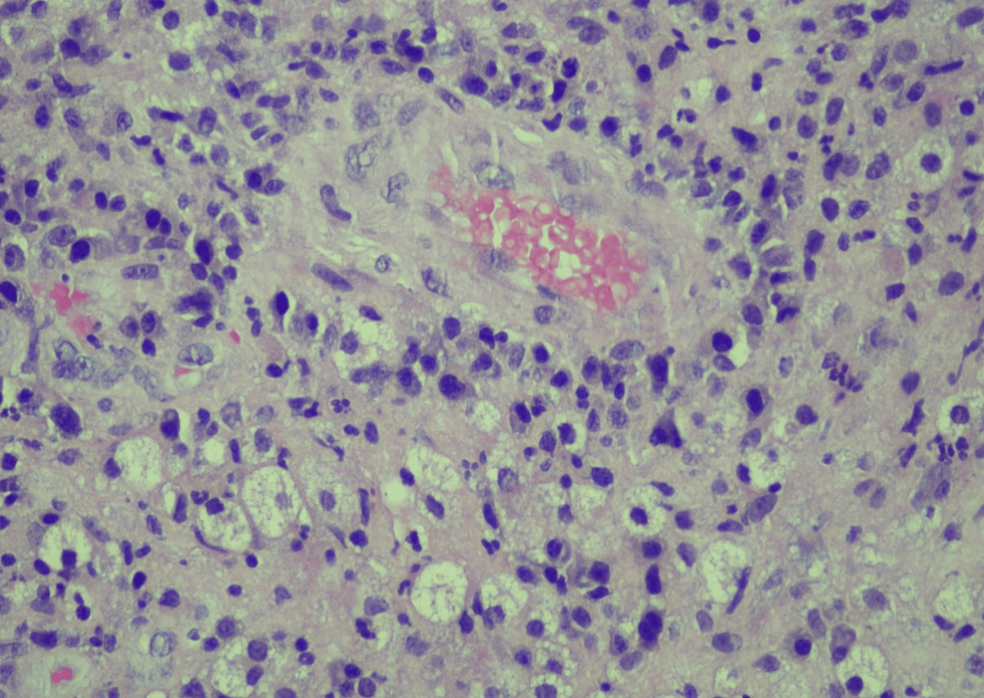
Photomicrograph of left breast core biopsy Hematoxylin and eosin staining original magnification x 40; showing blood vessels surrounded by lymphocytes, epitheloid histiocytes, spindle cells, and foamy macrophages.

**Figure 4:**
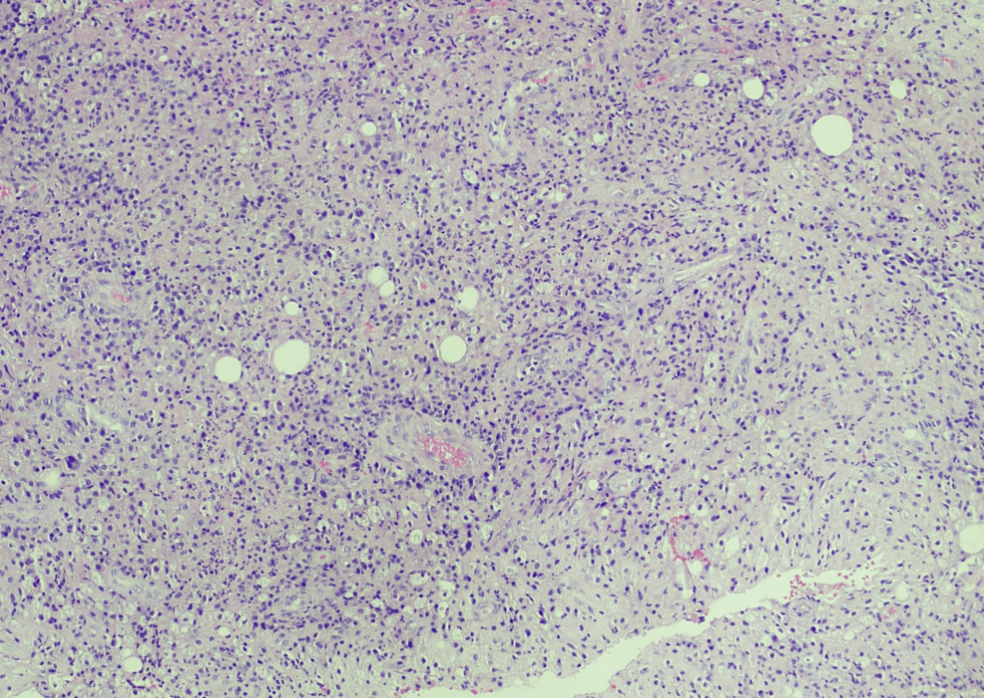
Photomicrograph of left breast core biopsy Hematoxylin and eosin staining original magnification x20, highlighting acute and chronic inflammatory cells including lymphocytes, epitheloid histiocytes, spindle cells, plasma cells, and fat cells surrounded by inflammation.

All five patients presented with a palpable mass in the breast. Case 5 had chronic changes with abscesses and fistula formation (Figures 1, 2); all had positive biopsy results (Figures 3, 4) confirming granulomatous mastitis. Out of the five patients, two were lactating mothers who had recently given birth, and two had other autoimmune conditions like erythema nodosum or alopecia. All five patients initially consulted a breast surgeon, and two of them required incision and drainage. All patients received antibiotics and nonsteroidal treatment before being referred to rheumatology for further management. The rheumatology treatment plan included corticosteroids with or without methotrexate and close follow-up, refer to Table 1 for a detailed description of each case.

**Table 1:** Case reports BI-RADS: Breast Imaging-Reporting and Data System

	Clinical presentation	Initial management.	Subsequent management	Imaging	Other auto-immune conditions	Histopathology	Outcome
Case 1 80-year-old Female.	Left breast mass	Doxycycline + Celebrex for 1 month	None	Ultrasound: Complex cyst with irregular borders surrounded by hypoechogenic tissue and edema. Mammogram - prominent 5.5 x 6.6 x 5.6 cm mass with irregular margin, no suspicious microcalcifications. Risk assessment using the Tyrer-Cuzick model - lifetime risk of developing breast cancer 1.5 %.	N/A	Core biopsy: Granulomatous mastitis. Acute and chronic inflammatory cells with lymphocytes, histiocytes, plasma cells, and foamy macrophages. Pan Cytokeratin immunostain x4 negative for infiltrating epithelial elements. Acid-fast Bacilli (x1) and Gram stain (x1) stains are negative for microorganisms and Negative for atypia/malignancy (Figures 1, 2).	Complete remission
Case 2 28-year-old Female	Right breast mass	Incision and drainage. Pathology confirms IGM	Doxycycline 100 mg BID+ Celebrex x 30 days Prednisone 10 mg (for >6 months)	Bilateral diagnostic mammogram - heterogeneous dense 9 cm oval mass with ill-defined margins encompassing the right upper outer breast. BI-RADS 4 - suspicious for malignancy. Recommend ultrasound core-guided biopsy. Right breast ultrasound showing dense breast shadowing of the right upper outer breast noted without discernible mass. Axilla: lymph nodes demonstrate mild cortical thickening up to 0.4cm. Recommend core guided biopsy. Repeat, right breast ultrasound showed, heterogeneous echotexture throughout with regions of irregular hypodensity predominantly in lateral breast with findings consistent with granulomatous mastitis No evidence of abscess BI-RADS 2-benign.	Erythema Nodosum	Core biopsy - breast with multiple microabscesses within several of the ducts and lobular area. The stroma in places with prominent fibro hyalinized changes. Incision & drainage (I&D) pathology multiple microabscesses associated with granulomatous multinucleated giant cell reaction.	Relapse in 30 days when treatment was discontinued then remission achieved after 6 months of prednisone treatment
Case 3 31-year-old pregnant 30 weeks	Right breast abscess	Incision and drainage x 2	Prednisone 20 mg x 7 days, followed by prednisone 10 mg x 5 weeks.	US breast - multiple hypoechoic masses (largest 4cm ) in the right lower quadrant, may represent granulomatous mastitis or chronic abscess. BI-RADS 4	Alopecia	Biopsy - breast tissue with clusters and pools of polymorphonuclear neutrophils consistent with abscess and single focus of epitheloid inflammation.	Complete remission
Case 4 24-year-old Female	Left breast mass	A 30-day course of both doxycycline and Celebrex reports partial improvement in pain, redness, and drainage from the fistula tract.	Prednisone 20 mg -partial response methotrexate + Folic Acid 10 mg weekly for one month. The methotrexate dose was increased to 15 mg Q weekly for 8 months.	Left breast ultrasound 7.9 cm area of hypoechogenicity in the 8:00-12:00 breast may represent abscess vs granulomatous mastitis. Left axillary adenopathy.	N/A	Left breast U/S-guided aspiration and biopsy. Extensive acute and chronic inflammatory changes with granulomatous reaction. Cultures negative.	Complete remission. Treatment discontinued after 8 months of treatment as described.
Case 5 32-year-old Female	Right breast pain and swelling (Figures 3, 4)	3 weeks of doxycycline for 7 days - partial improvement 4 months later - Incision and drainage.	Doxycycline + Celebrex x 30 days + Prednisone + Azathioprine.	US right breast shows one 1.5 x 1.2 cm hypoechoic nodule at 7 to 8 o’clock position and, a second heterogenous 1.2 x 0.5 x 0.6 cm nodule at 7 to 8’o clock position. BI-RADS 3 (benign). Mammography- multilobulated mass-like area 6 x 4.5 cm with no suspicious microcalcifications or distortion.	N/A	Right US-guided biopsy - acute inflammation with abscess formation, granulation tissue, and few multinucleated giant cells present within the inflammatory reaction.	Partial remission

Results

All of the patients received initial treatment with antibiotics, mainly doxycycline. In addition to antibiotics, celecoxib was given to 80% (Cases 1, 2, 4, 5) of the patients for a minimum of three weeks. Among these patients, 60% (Cases 2, 4, 5) had a relapse after three weeks and required repeat treatment with antibiotics and additional treatment. Twenty percent (20%) (Case 3) received only corticosteroids. Twenty percent (20%) (Case 1) of the patients achieved full remission of the disease.

Four out of five patients were treated with prednisone starting at a dose of 20 mg and tapered down to 10 mg for an average of five months. All four patients experienced remission within an average of three months. Of the patients who received prednisone, 25% experienced relapse after discontinuation. An additional agent, methotrexate 10 mg Q weekly, was given to 25% of the patients. 

Two of the five patients had incision and drainage (I&D) of the mass before any treatment was initiated. One patient had an I&D after treatment was started.

Out of the five patients studied, one was a 30-week pregnant woman (Case 3), one was an 80-year-old healthy female (Case 1), and the other three were between the ages of 24 and 32. Overall, only two out of five (⅖) patients experienced complete resolution at six months from all treatments. One out of five (⅕) continued to have a persistent condition and two out of five (⅖) required prolonged treatment/addition of immunosuppressive agents to achieve remission.

## Discussion

We conducted a literature review, to identify articles on granulomatous mastitis and therapeutic approaches, we did a PubMed search using the Mesh terms "Granulomatous Mastitis/drug therapy" OR "Granulomatous Mastitis/surgery" OR "Granulomatous Mastitis/therapy". A total of 224 articles were found. We included all full-length case series and retrospective and cohort studies but excluded studies describing patients with granulomatous mastitis due to tuberculosis or other infectious causes, papers not published in English, papers that focused on invasive procedures, and descriptive papers with no intervention or outcome. Ten papers were included in the final analysis.

Many autoimmune diseases may affect breast tissue [12], including IGM, which is a specific autoimmune disease that primarily affects young women of childbearing age. However, our case series has shown evidence of IGM in an elderly female as well. IGM can often present symptoms similar to breast cancer, which is why patients undergo invasive procedures to receive a prompt diagnosis and treatment. Two of our patients also had other extramammary manifestations, such as erythema nodosum and alopecia, therefore all patients diagnosed with IGM should also be tested to rule out other underlying autoimmune diseases and infectious diseases, such as tuberculosis.

In terms of initial diagnostic testing, patients typically undergo radiological studies such as breast ultrasound and mammography [13]. A BI-RADS 2 or 3 diagnosis does not always require invasive procedures like surgical resection. However, most patients undergo confirmatory core-needle biopsies. Histologically, the results are consistent across all cases, with predominantly inflammatory granulomatous changes and negative cultures.

Clinicians have been perplexed by the chronic nature and unclear cause of IGM, making their management a complex task. This discussion focused on the challenges faced in diagnosing such a disease, the lack of standardized treatments available, and potential avenues for future research. Current literature reveals a wide range of treatment responses, leading to significant heterogeneity.

Our case series included only Hispanic patients, confirming that this is a common entity in this patient population [12].

There is no standardized treatment protocol for IGM, various modalities have been used such as antibiotics, surgical excision, and immunosuppressive therapy with methotrexate, celecoxib, and corticosteroid. However, prednisone has been shown to achieve the longest remission periods in 69% of the cases followed by surgery in 65 % [14].

## Conclusions

There is scope for further research to understand the underlying mechanisms that trigger inflammatory responses leading to IGM. It is also important to investigate risk factors, such as environmental, genetic, and immune-mediated, to categorize and personalize management strategies. Additionally, a large-scale multidisciplinary collaboration to create a comprehensive registry could help in understanding the epidemiology of the disease in the future.
